# Supplementary material for: Effectiveness of the e-NurSus Children Intervention in the Training of Nursing Students
Source: Int J Environ Res Public Health. 2019 Nov 5;16(21):4288. doi: 10.3390/ijerph16214288 (PMC6862581; doi:10.3390/ijerph16214288)
Supplement: Supplementary file 1 [file ijerph-16-04288-s001.pdf]

Table S1. Means for attitudes and skills items and correct answers for knowledge items in the pre-test and post-test scales in Spain and United Kingdom

Item	Spain[1]				United Kingdom[2]			
	Pre-value[3]	Post-value[3]	% increase	*p*-value[4]	Pre-value[3]	Post-value[3]	% increase	*p*-value[4]
Attitudes	*M*	*M*			*M*	*M*		
1. Climate change is an important issue for nursing.	5.98 ± 1.27	6.80 ± 0.56	13.71%	<0.001	5.29 ± 1.41	6.22 ± 1.28	17.58%	<0.001
2. Issues about climate change should be included in the nursing curriculum.	4.64 ± 1.62	6.31 ± 0.92	35.99%	<0.001	4.74 ± 1.58	5.88 ± 1.34	24.05%	<0.001
3. Sustainability is an important issue for nursing.	5.49 ± 1.42	6.54 ± 0.81	19.13%	<0.001	6.08 ± 1.20	6.27 ± 1.18	3.03%	0.084
4. Sustainability should be included in the nursing curriculum.	4.69 ± 1.64	6.26 ± 0.98	33.48%	<0.001	5.68 ± 1.35	6.11 ± 1.67	7.57%	<0.001
5. I apply sustainability principles at home.	4.90 ± 1.46	5.69 ± 1.21	16.12%	<0.001	5.27 ± 1.39	5.57 ± 1.26	5.69%	0.011
Knowledge	Correct Answers	Correct Answers			Correct Answers	Correct Answers		
1. The pediatric population is more susceptible to environmental threats due to their biological immaturity.	103	110	6.80%	1.000	112	152	35.71%	<0.001
2. The increased energy and metabolic consumption of the pediatric population protects children from environmental hazards.	70	94	34.29%	0.001	66	84	27.27%	0.027
3. The higher rate of cell growth during the pediatric age increases the risk of health effects caused by environmental factors.	71	102	43.66%	<0.001	83	132	59.04%	<0.001
4. Environmental factors do not influence hormonal secretion during puberty.	95	106	11.58%	0.013	101	123	21.78%	0.002
5. Nitrogen oxide from fossil fuels in the home and tobacco smoke causes redness and burns on the skin.	14	8	-42.86%	0.238	27	20	-25.93%	0.311
6. Particles from animals exacerbate asthma crisis.	68	108	58.82%	<0.001	108	145	34.26%	<0.001
7. Increased humidity at home improves respiratory diseases in children.	59	95	61.02%	<0.001	69	74	7.25%	0.590
8. Passive smoking is associated with the development of acute leukemia in children.	51	98	92.16%	<0.001	67	120	79.10%	<0.001
9. Childhood leukemia incidence rates are higher in the areas most exposed to radon.	45	93	106.67%	<0.001	86	132	53.49%	<0.001
10. Overexposure to solar ultraviolet radiations can damage the skin of adults more severely than that of children.	83	88	6.02%	0.442	105	102	-2.86%	0.780
11. During childhood more than half of the expected lifetime solar ultraviolet radiation is absorbed.	51	95	86.27%	<0.001	50	98	96.00%	<0.001
12. Lead accumulates in the body affecting the nervous system.	77	100	29.87%	<0.001	81	127	56.79%	<0.001
13. Chronic dietary exposure to mercury (fish and shellfish) is less toxic to children's central nervous system than to adults.	66	94	42.42%	<0.001	90	119	32.22%	<0.001
14. Exposure to pesticides increases the risk of developing attention deficit problems in school-aged children.	53	94	77.36%	<0.001	54	111	105.56%	<0.001
15. Children born to smoking mothers during pregnancy are at risk of lower intellectual capacity.	63	95	50.79%	<0.001	96	133	38.54%	<0.001
16. Exposure to organic solvents during fetal development can cause learning disabilities in children.	63	96	52.38%	<0.001	66	111	68.18%	<0.001
17. Water containing nitrates can only cause intoxication during childhood.	73	72	-1.37%	1.000	64	94	46.88%	0.001
18. Chlorination of water forms sub-products from the disinfection process that have been classified as carcinogenic.	39	90	130.77%	<0.001	34	97	135.29%	<0.001
19. The major source of childhood exposure to pesticides is through ambient air.	19	11	-42.11%	0.115	26	20	-23.08%	0.362
20. The main route of exposure to mercury is through cereal intake.	45	42	-66.67%	0.766	74	63	-14.86%	0.185
21. Exposure to lead through diet occurs mainly through fish intake.	22	23	4.55%	1.000	27	42	55.56%	0.036
22. Food colourings and preservatives are associated with central nervous system problems.	37	89	140.54%	<0.001	40	85	112.50%	<0.001
23. Genetically modified foods cause fewer allergic reactions in children.	67	87	29.85%	0.002	73	86	17.81%	0.136
24. Schools and nurseries are environmentally safe places.	97	105	8.24%	0.057	75	122	62.67%	<0.001
25. Children are exposed to higher concentrations of air pollutants at home than outdoors.	61	65	6.56%	0.678	78	76	-2.56%	0.905
26. Parks and gardens are the areas with the least environmental pollutants where children can play.	67	85	26.87%	0.015	68	86	26.47%	0.036
Skills	*M*	*M*			*M*	*M*		
1. I am able to assess the main environmental risks to which a child is exposed.	3.10 ± 0.93	4.27 ± 0.60	37.74%	<0.001	3.36 ± 0.90	4.01 ± 0.83	19.35%	<0.001
2. I am NOT able to identify the environmental risks that can cause respiratory diseases in a child.	3.18 ± 1.01	4.26 ± 0.82	33.96%	<0.001	3.66 ± 0.90	4.04 ± 0.88	10.38%	<0.001
3. I am able to identify the environmental risks that can cause neoplastic diseases in a child.	2.72 ± 0.90	4.20 ± 0.83	54.41%	<0.001	2.02 ± 0.94	3.24 ± 1.10	60.40%	<0.001
4. I am NOT able to identify the environmental risks that can cause neurological disorders in a child.	2.92 ± 1.12	4.20 ± 0.75	43.84%	<0.001	2.75 ± 1.07	3.55 ± 0.98	29.09%	<0.001
5. I am able to provide health education to parents about the main contaminants in their child's food.	2.76 ± 1.19	4.33 ± 0.90	56.88%	<0.001	2.61 ± 1.09	3.64 ± 0.96	39.46%	<0.001
6. I am NOT able to identify the environmental risks in playgrounds.	3.08 ± 1.06	4.17 ± 0.72	35.39%	<0.001	3.40 ± 1.01	3.93 ± 0.89	15.59%	<0.001
7. I am able to provide health education to parents about actions to minimize environmental risks to which a child is exposed when playing outdoors.	2.80 ± 1.06	4.39 ± 0.93	56.79%	<0.001	3.11 ± 0.98	3.81 ± 0.86	22.51%	<0.001
8. I am NOT able to identify the environmental risks in a child's home.	3.06 ± 0.94	4.31 ± 0.66	40.85%	<0.001	3.67 ± 0.90	4.07 ± 0.88	10.90%	<0.001
9. I am able to provide health promotion to parents about environmental risks at home.	2.92 ± 1.02	4.25 ± 0.79	45.56%	<0.001	3.45 ± 0.96	4.04 ± 0.78	17.10%	<0.001
10. I am able to identify the environmental risks in a child's school.	3.01 ± 0.89	4.27 ± 0.91	41.86%	<0.001	3.34 ± 1.02	3.93 ± 0.79	17.66%	<0.001
11. I am NOT able to identify the actions needed to combat environmental risks in a child's school.	3.09 ± 1.02	4.23 ± 1.00	36.89%	<0.001	3.20 ± 0.96	3.83 ± 0.89	19.69%	<0.001
12. I do NOT feel able to do my job as a nurse in a Pediatric Environmental Health Specialty Unit.	2.83 ± 1.22	4.27 ± 0.60	50.88%	<0.001	3.01 ± 1.02	3.68 ± 0.97	22.26%	<0.001

[1] n = 110 for attitudes, n = 110 for knowledge and n = 109 for skills. [2] n = 157 for attitudes, n = 157 for knowledge and n = 154 for skills. [3] Means for attitudes and skills items and correct answers for knowledge items. [4] *p*-value in the Wilcoxon test for ordinal variables (attitudes and skills items) and McNemar test for nominal variables (knowledge items) comparing pre-test with post-test results.
